# Exposure to PAHs during Firefighting Activities: A Review on Skin Levels, In Vitro/In Vivo Bioavailability, and Health Risks

**DOI:** 10.3390/ijerph191912677

**Published:** 2022-10-04

**Authors:** Gabriel Sousa, Joana Teixeira, Cristina Delerue-Matos, Bruno Sarmento, Simone Morais, Xianyu Wang, Francisca Rodrigues, Marta Oliveira

**Affiliations:** 1 REQUIMTE/LAQV, Instituto Superior de Engenharia, Instituto Politécnico do Porto, R. Dr. António Bernardino de Almeida 431, 4249-015 Porto, Portugal; 2 CESPU-Instituto de Investigação e Formação Avançada em Ciências e Tecnologias da Saúde, Instituto Universitário de Ciências da Saúde, 4585-116 Gandra, Portugal; 3 Instituto de Investigação e Inovação em Saúde, Universidade do Porto, 4200-135 Porto, Portugal; 4 QAEHS-Queensland Alliance for Environmental Health Sciences, The University of Queensland, Woolloongabba, QLD 4102, Australia

**Keywords:** dermal exposure, firefighters, polycyclic aromatic hydrocarbon, fire combat, dermal absorption, skin diseases

## Abstract

Occupational exposure as a firefighter is a complex activity that continuously exposes subjects to several health hazards including fire emissions during firefighting. Firefighters are exposed to polycyclic aromatic hydrocarbons (PAHs), known as toxic, mutagenic, and carcinogenic compounds, by inhalation, dermal contact, and ingestion. In this work, a literature overview of firefighters’ dermal exposure to PAHs after firefighting and data retrieved from skin in vitro/in vivo studies related to their dermal absorption, bioavailability, and associated toxicological and carcinogenic effects are reviewed. The evidence demonstrates the contamination of firefighters’ skin with PAHs, mainly on the neck (2.23–62.50 ng/cm^2^), wrists (0.37–8.30 ng/cm^2^), face (2.50–4.82 ng/cm^2^), and hands (1.59–4.69 ng/cm^2^). Concentrations of possible/probable carcinogens (0.82–33.69 ng/cm^2^), including benzopyrene isomers, were found on firefighters’ skin. PAHs penetrate the skin tissues, even at low concentrations, by absorption and/or diffusion, and are locally metabolized and distributed by the blood route to other tissues/organs. Lighter PAHs presented increased dermal permeabilities and absorption rates than heavier compounds. Topical PAHs activate the aryl hydrocarbon receptor and promote the enzymatic generation of reactive intermediates that may cause protein and/or DNA adducts. Future research should include in vitro/in vivo assays to perform a more realistic health risk assessment and to explore the contribution of dermal exposure to PAHs total internal dose.

## 1. Introduction

Human exposure to air pollution is recognized as the single biggest threat to human health by causing millions of deaths and loss of healthy life annually [1]. Human environmental and occupational exposure to airborne pollutants through inhalation have been extensively characterized over the last decades [2,3,4,5,6]. At the interface with the air, skin, the largest organ of the human body, is the first-line barrier of protection against health-hazardous compounds. The dermal contact with pollutants may activate cell metabolism and promote oxidative stress and inflammatory processes; however, their impact on skin remains poorly explored [7,8,9,10,11].

Skin comprises three layers, namely, the epidermis, dermis, and hypodermis, and its normal barrier function can be altered through a disturbance of lipid metabolism at the surface layers. This organ can be damaged by exposure to different external factors, such as solar ultraviolet (UV) radiation, microorganisms, dehydration, and air pollutants (e.g., particulate matter, volatile organic compounds including polycyclic aromatic hydrocarbons (PAHs), carbon monoxide, and ozone) [7,8,9,11,12]. After dermal contact, air pollutants can be absorbed and/or dissolved on the outermost layer of the epidermis containing different proportions of sebum and sweat. Keratinocytes represent more than 90% of the epidermis cells and have an important role in antioxidant defense, since they can enzymatically metabolize organic pollutants, being directly involved in the response to dermal toxicity and inflammation [13,14,15]. The dermis and hypodermis can also be contaminated with pollutants assimilated by other routes of exposure, e.g., inhalation and ingestion, and afterwards, xenobiotics are transported within the blood system (systemic contamination) and distributed to the different tissues and organs [7].

PAHs are formed during the incomplete combustion of organic matter and can be released from natural (e.g., volcanoes and forest fires) and anthropogenic (e.g., traffic emissions, production of energy from fossil fuels, agricultural activities, heating, and cooking) sources [16]. These health-relevant persistent organic pollutants are well known for their toxic, carcinogenic, and mutagenic properties [17,18,19]. PAHs are lipophilic compounds, particularly those with high molecular weight, and they tend to accumulate in fat tissues. Besides this, PAHs are also known as potential endocrine disruptors [16,20]. The exposure to PAHs has been associated with reproductive, developmental, and hemato-, cardio-, neuro-, and immunotoxicity effects [16]. Evidence related to the interaction between PAHs and the skin has been slowly emerging during the last decade. The normal barrier function of the skin can be affected by disorders on the lipid metabolism in the stratum corneum layer, dysfunction of the deoxyribonucleic acid (DNA), and/or disorders of the skin’s protein composition [8]. Dermal contact with PAHs represents an additional risk to skin tissues; however, the link between topical concentrations and cutaneous inflammation remains poorly characterized [13,21,22]. PAHs can permeate the skin barrier and promote the formation of reactive oxygen species (ROS), which stimulate oxidative stress, being responsible for the development and/or exacerbation of inflammatory skin diseases (e.g., atopic and contact dermatitis, eczema, psoriasis, acne, and skin aging) [7,8,9,11,21]. The formation of ROS is triggered by the aryl hydrocarbon receptor (AhR)-dependent induction of CYP1 isoforms, since PAHs are AhR activators [9,10,23]. During the enzymatic metabolism of PAHs on topical tissues, different intermediate compounds, such as epoxides and diols, are formed [24]. These reactive intermediates can induce mutagenesis and the formation of tumors by binding to cell proteins and DNA, which might contribute to the development of skin cancer [8,9]. Some authors already described a synergetic effect between the dermal exposure to PAHs and UV radiation and the acceleration of skin diseases, including cancer [7,25].

In June 2022, firefighters’ occupational exposure to PAHs was classified as known carcinogen to humans (Group 1) by the International Agency for Research on Cancer (IARC) [26]. The available literature demonstrated sufficient evidence for the development of mesothelioma and bladder cancer in humans and limited evidence for the development of melanoma, colon, prostate, and testicular cancers, and non-Hodgkin’s lymphoma [26,27,28]. During fire combat, firefighters are regularly exposed to PAHs, known/possible/probable carcinogens [18,19], via inhalation, dermal contact, and ingestion [28,29,30,31,32,33]. Dermal contact with the PAHs released from fires occurs not only during firefighting, despite the correct use of personal protective equipment (PPE), but also during the donning and/or doffing of PPE and contact with contaminated firefighting materials and vehicles [30]. Information has been slowly emerging during the last decade [29,34,35,36,37,38,39]. Despite the contamination of firefighters´ skin with PAHs released from firefighting being demonstrated, scarce information is available relating to the transcutaneous penetration of PAHs, dermal bioaccessibility and bioavailability, and the consequent health risks. Therefore, this work aims to: (i) extract data relating to dermal exposure to PAHs after firefighting events from the available studies, and (ii) collect the information retrieved from dermal in vitro/in vivo studies that determine parameters conditioning the topical uptake of PAHs, rates of permeability, bioavailability, and toxic and carcinogenic effects after dermal exposure to PAHs. Further studies concerning this issue should be addressed, since a broad multidisciplinary knowledge is urgently needed to better understand the topical assimilation of PAHs and the metabolic processes that occur within skin tissues. These studies will generate knowledge that would contribute to a more realistic health risk assessment, to define the critical levels for firefighters’ dermal exposure, and to evaluate the efficiency of the available dermal preventive measures.

## 2. Methods

A literature search was conducted using several online databases: ISI Web of Knowledge, Science Direct, PubMed, Google Scholar, Scopus, and Scielo. Occupational exposure, firefighters, dermal exposure, PAHs, skin in vitro/in vivo studies, bioavailability, and bioaccessibility were the keywords used in the search. All of the available studies published up to 2021 were considered. In total, 13 studies reporting the concentrations of PAHs on the skin of firefighters after firefighting were selected for analysis. Regarding the in vivo/in vitro studies, all of the reports that described the dermal exposure to PAHs using animal and/or human skin models were considered. A total of 19 studies were retrieved from the literature, with eight characterizing dermal exposure to PAHs, four addressing bioavailability, and seven concerning toxicological and carcinogenic potential of PAHs on skin tissues.

## 3. Concentrations of PAHs on the Skin of Firefighters

### 3.1. Levels of Total PAHs

A total of 13 studies assessed the firefighters’ dermal exposure during firefighting activities at structural, controlled, and training fire exercises; only two studies evaluated skin contamination from live fire scenarios [34,40]. However, one study [34] presented data in µg/wipe, a concentration unit that is not comparable with the results reported in other studies. The available studies characterized the exposure of Nordic (50%), North American (42%), and Australian (8%) firefighters and represented periods of exposure to fire emissions up to 30 min. The eligible studies evaluated skin contamination on the neck (*n* = 7), wrist and/or arm (*n* = 4), hand, including fingers (*n* = 4), face and/or forehead (*n* = 2), calf (*n* = 1), scrotum (*n* = 1), and back (*n* = 1) of firefighters.

Overall, the concentrations of total PAHs on the skin of firefighting forces ranged from 4.00 ng/cm^2^, in a Swedish study with 20 subjects during training exercises [37], to 1200 ng/cm^2^, during training activities performed by 19 firefighters at a fire house containing conifer plywood [41]. Keir et al. [36] reported significantly increased concentrations of total PAHs (pre- to post-exposure change of 3.3 times: 0.5 versus 1.6 ng/cm^2^) on the neck, wrist, and forehead skin of 28 firefighters, after the suppression of an emergency fire. Sjöström et al. [29] determined the levels of PAHs on the skin of the wrists and neck of seven firefighters performing three different roles in fire events: team leaders responsible for the supervision of fire simulations, who stood far from the fire line; fire starters who initiated the fire exercise; and firefighters who fought the fire. The authors found significantly increased concentrations of total PAHs on the skin of the fire starters (20.42 ng/cm^2^) than on the skin of the smoke divers (7.39 ng/cm^2^) and team leaders (7.00 ng/cm^2^). In the study performed by Andersen et al. [40], nine firefighters were enrolled in small emergency fire calls to an outdoor fire in a car and a fire in an electrical installation of a basement. The level of skin exposure to total PAHs reported after the events was, on average, 18.1 ng/cm^2^; no differences were observed between pre- and post-shift levels. In 2014, Fent and coworkers reported lower concentrations of total PAHs on the skin regions (arm, neck, scrotum, hand, and face) of subjects participating in round 1 compared to those participating in round 2 controlled structural burns [35]. In that study, the round 1 fire burns were slightly longer than in round 2, and distinct fire scenarios were considered. In the round 1 fire scenario, the firefighters were stagnant, and the timber-framed room with a drywall interior was more than the double that of the round 2 metallic room. These changes contributed to different fire emissions and a less-concentrated smoke layer was observed in the first round. Moreover, subjects of the second round of controlled burns used brand new hoods that were approximately 7.5 cm longer [35]. In another study, Laitinen et al. [41] evaluated the dermal exposure of 19 firefighters during fire training exercises with a sampler located under the PPE and on the surface of the chest and the back skin of participants. The authors reported mean values of dermal total PAHs of 1200 ng/cm^2^ for the fire house containing conifer plywood, 760 ng/cm^2^ for the exercise in the container with conifer plywood, and 30 ng/cm^2^ in event with a gas simulator.

**Figure 1:**
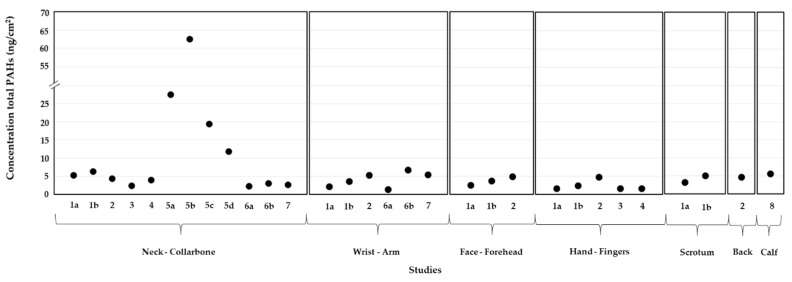
Concentrations of total PAHs on the skin of firefighters after firefighting activities (1—Fent et al. [35] (a—Round 1 fire; b—Round 2 fire); 2—Fernando et al. [45]; 3—Fent et al. [44]; 4—Wingfors et al. [37]; 5—Andersen et al. [43] (a—Campaign 1; b—Campaign 2; c—Campaign 3; d—Campaign 4); 6—Banks et al. [42] (a—diesel pan fire; b—particleboard fire); 7—Strandberg et al. [46]; 8—Beitel et al. [32]).

Figure 1 presents the levels of total PAHs on different skin regions of firefighters after firefighting. The evidence demonstrates that the neck/collarbone was the most characterized body site, with concentrations ranging from 2.23 ng/cm^2^, during a 15 min controlled compartment fire with diesel [42], to 62.50 ng/cm^2^, in a training exercise lasting up to 10 min [43] (Figure 1). Some authors reported increased values of total PAHs on the neck of exposed firefighters compared to other body regions [35,37,42,44]. Moreover, Fent et al. [35] concluded that neck is the most likely region to be contaminated with fire emissions, since other body parts are usually covered with multiple layers of clothes and/or the PPE. However, in a Swedish study performed by Sjöström et al. [29], higher values of PAHs were found on the wrist skin compared with the neck, across all of the firefighters participating on a training exercise: 16.07 versus 4.35 ng/cm^2^ for fire starters, 4.25 versus 3.14 ng/cm^2^ for the smoke divers, and 4.69 versus 2.31 ng/cm^2^ for the team leaders. Andersen et al. [43] reported the highest concentrations of total PAHs on the necks of firefighters during different training exercises, principally during Campaigns 1 and 2 with the burning of wood pallets, while Campaigns 3 and 4 contained the burning of electrical cords and mattresses (Figure 1). Banks et al. [42] reported lower concentrations of PAHs (2.23 versus 3.06 ng/cm^2^) on the neck skin of Australian firefighters participating in a diesel-based fire than in a wood conglomerate simulated fire. The authors attributed the observed differences to the composition of the chipboard material burned: wood particles (>85%), glued with melamine/urea/formaldehyde resin (<13%), paraffin wax (<2%), and formaldehyde (0.0001%) that might release higher levels of PAHs. Similar results were also reported by Wingfors et al. [37].

The levels of total PAHs were also reported on the wrist/arm (0.37 to 8.30 ng/cm^2^), face/forehead (2.50 to 4.82 ng/cm^2^), and hands/fingers (1.59 to 4.69 ng/cm^2^) of exposed firefighters (Figure 1). Only one study assessed the levels of PAHs on the skin of the scrotum of 15 firefighters involved in two controlled fires that lasted between 15 and 30 min [35]. The concentrations of total PAHs on the skin of the scrotum were increased in round 2 (5.04 ng/cm^2^; intermodal metal room with 15 m^3^ and mobile firefighters) than in round 1 (3.14 ng/cm^2^; 33 m^3^ timber-framed burn room and stationary subjects) (Figure 1). Moreover, the authors observed increased levels of PAHs on the skin of the neck, wrist, face, and hands of firefighters involved in the burns of round 2 (Figure 1). A study performed by Fernando et al. [45] with 28 volunteer firefighters attending a fire with untreated wood (≈30 min) reported total PAH values of 4.58 ng/cm^2^ on the skin of the back (Figure 1). Beitel et al. [32] determined the levels of total PAH (mean of 5.58 ng/cm^2^) on the calf of 11 subjects involved in a controlled indoor fire that lasted 14 min.

Among all of the PAHs reported in the available literature, phenanthrene, fluoranthene, and pyrene were the main contributors to the total PAH concentrations found on the skin of the firefighters, regardless of the type of fire and the body site monitored [29,37,42,43,45].

### 3.2. Levels of Pyrene

**Figure 2:**
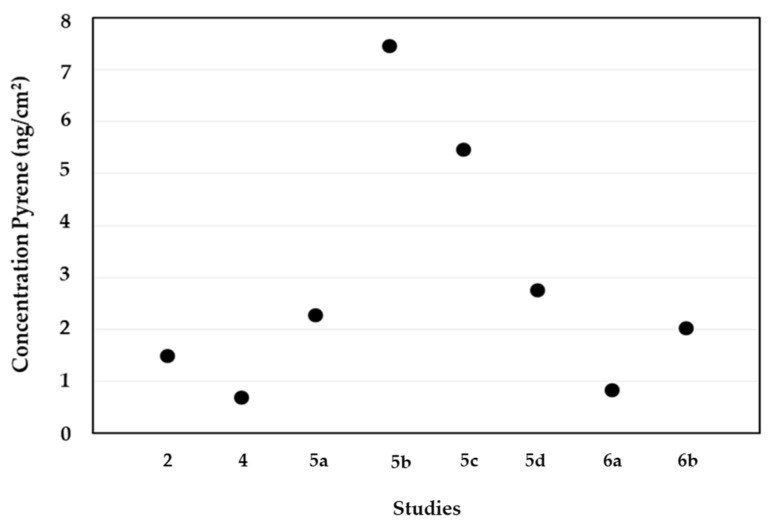
Concentrations of pyrene on the skin of firefighters after firefighting activities (2—Fernando et al. [45] (total of neck, wrist, back, forehead, and fingers); 4—Wingfors et al. [37] (neck); 5—Andersen et al. [43] (a—Campaign 1; b—Campaign 2; c—Campaign 3; d—Campaign 4) (neck); 6—Banks et al. [42] (a—diesel pan fire; b—particleboard fire) (total of neck and wrist)).

Figure 2 presents the concentrations of pyrene, a biomarker of PAHs exposure, determined on the skin of firefighters after fire combat. Overall, the total levels of pyrene on the skin ranged between 0.70 ng/cm^2^, in a training exercise with 20 Swedish firefighter students [37], and 7.46 ng/cm^2^, in the second campaign of a training exercise with 53 Danish firefighters [43]. Banks et al. [42] determined the concentrations of pyrene on the skin of the neck and wrist of 25 subjects after firefighting at simulated compartment fires. Skin contamination with pyrene was lower after a diesel-based fire than after a particleboard fire event (Figure 2). Moreover, the reported wrist values of pyrene (1.3 ng/cm^2^) were significantly higher at the particleboard fire than the levels (0.10 ng/cm^2^) determined during the diesel pan fire; no differences were observed in the concentrations of pyrene on the neck skin (0.74 ng/cm^2^) [42]. Sjöström et al. [29] reported increased levels of pyrene on the skin of fire starters (total of 3.68 ng/cm^2^, 2.97 on the wrists and 0.71 ng/cm^2^ on the neck) than for fire combatants (1.25 ng/cm^2^: 0.76 and 0.49 ng/cm^2^) and team leaders (1.09 ng/cm^2^: 0.78 and 0.31 ng/cm^2^) after participation in training exercises. Fernando et al. [45] assessed the levels of pyrene in five different skin regions of 28 firefighters (total of 1.50 ng/cm^2^) involved in fire training exercises with untreated wood (Figure 2). The authors found that the skin on the fingers presented the highest levels of pyrene (0.41 ng/cm^2^), followed by the neck (0.34 ng/cm^2^), forehead (0.30 ng/cm^2^), back (0.23 ng/cm^2^), and wrist (0.22 ng/cm^2^).

### 3.3. Levels of Carcinogenic PAHs

**Figure 3:**
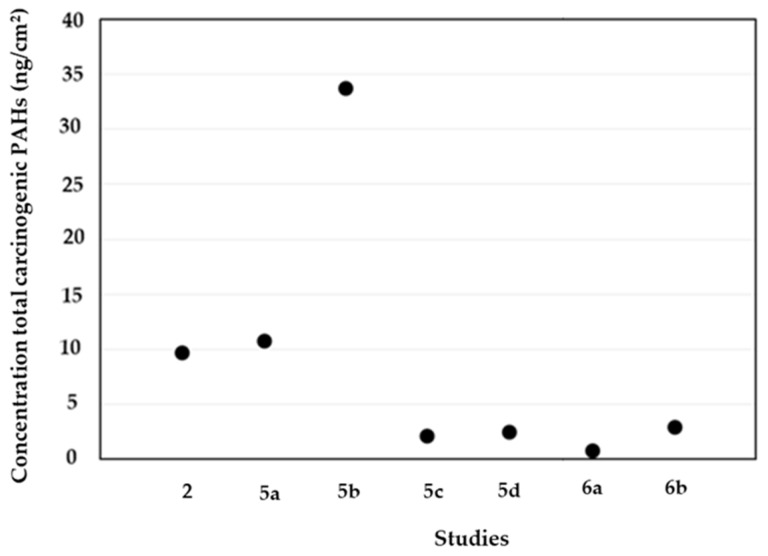
Concentrations of total carcinogenic PAHs (benzo(a)pyrene, naphthalene, benz(a)anthracene, chrysene, benzo(b)fluoranthene, benzo(j)fluoranthene, benzo(k)fluoranthene, benzo(e)pyrene, indeno(1,2,3-c,d)pyrene and dibenzo(a.h)anthracene) on the skin of firefighters after firefighting activities (2—Fernando et al. [45] (neck, wrist, back, forehead, and fingers); 5—Andersen et al. [43] (a—Campaign 1; b—Campaign 2; c—Campaign 3; d—Campaign 4) (neck); 6—Sjöström et al. [29] (neck); 6—Banks et al. [42] (a—diesel pan fire; b—particleboard fire) (neck and wrist)).

Benzo(a)pyrene is the only PAH classified as a known carcinogen to humans by the IARC [18]. The same agency classified some PAHs, namely, naphthalene, benz(a)anthracene, chrysene, benzo(b)fluoranthene, benzo(j)fluoranthene, benzo(k)fluoranthene, benzo(e)pyrene, indeno(1,2,3-c,d)pyrene, and dibenz(a.h)anthracene, as possible/probable carcinogens to humans [18,19]. The available studies included the abovementioned compounds, with exception of Fent et al. [35] (due to some matrix interferences caused by the use of corn oil at sampling collection). However, only some authors reported the individual concentrations of possible/probable carcinogenic PAHs [29,42,43,45]. Figure 3 presents the concentrations of total carcinogenic PAHs on the skin regions of firefighters after fire combat. Overall, the concentrations ranged from 0.82 ng/cm^2^, on the skin of 25 Australian firefighters attending a controlled diesel pan fire, to 33.69 ng/cm^2^, in the second campaign of a fire with wood pallets (Figure 3).

Banks et al. [42] presented lower values of carcinogenic PAHs on the skin (neck and wrist, respectively) of firefighters after a diesel fire (0.69 ng/cm^2^ and 0.13 ng/cm^2^) than at a particleboard fire (2.28 ng/cm^2^ and 0.81 ng/cm^2^). In a Swedish report, Sjöström et al. [29] assessed the levels of carcinogenic PAHs on the neck and wrist skin of firefighters after their participation in a training exercise. The participants of the study were tested while acting as fire starters, fire combatants, and team leaders (standing outside the burning house). According to the reported findings, the fire starters presented increased skin levels of carcinogenic PAHs compared to the other firefighters (2.84 ng/cm^2^ versus 1.16 ng/cm^2^ in fire combatants and 0.92 ng/cm^2^ in team leaders), which can be attributed to their increased proximity to the fire front [29]. In addition, the authors observed that the wrist skin presented levels of carcinogenic PAHs that were more than five times higher than the neck skin for fire starters (2.40 versus 0.44 ng/cm^2^, respectively), almost three times higher for smoke divers (0.86 versus 0.30 ng/cm^2^, respectively), and two times higher for team leaders (0.62 versus 0.30 ng/cm^2^, respectively). Overall, it was concluded that fire starters’ wrists were the most contaminated skin region [29]. Fernando et al. [45] reported higher concentrations of carcinogenic PAHs on the forehead skin (2.16 ng/cm^2^), followed by the back (2.07 ng/cm^2^), the wrist (1.87 ng/cm^2^), the fingers (1.81 ng/cm^2^), and the neck (1.76 ng/cm^2^).

**Figure 4:**
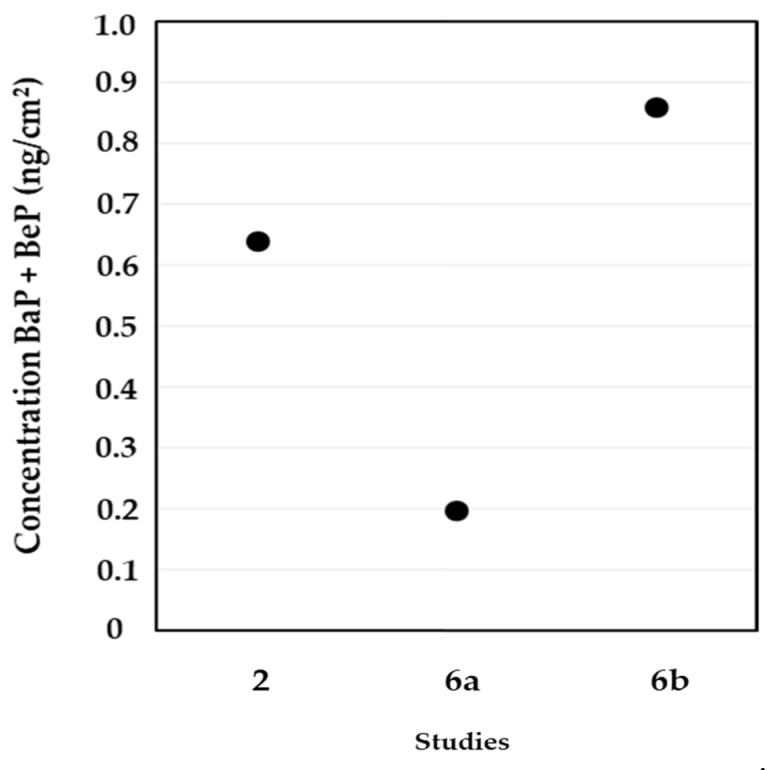
Concentrations of benzo(a)pyrene and benzo(e)pyrene on the skin of firefighters after firefighting activities (2—Fernando et al. [45] (neck, wrist, back, forehead and fingers); 6—Banks et al. [42] (a—diesel pan fire; b—particleboard fire) (neck and wrist)).

Only three studies were able to detect benzopyrene isomers, benzo(a)pyrene and benzo(e)pyrene, on the skin of the neck, wrists, forehead, fingers, and back of firefighters [29,42,45]. Recently, Banks and coworkers [42] reported significantly increased levels of benzopyrene isomers on the skin of the neck and wrists of Australian firefighters after particleboard simulated fires compared to diesel pan burns (four times higher: 0.86 versus 0.20 ng/cm^2^) (Figure 4). Regarding the skin regions studied, the authors also found higher concentrations of benzopyrene isomers on the skin of the wrists (0.62 ng/cm^2^) than on the neck (0.23 ng/cm^2^) after the wood conglomerate fire. However, contrary results were obtained from the diesel fire simulation (0.027 ng/cm^2^ in the wrists versus 0.17 ng/cm^2^ in the neck) [42]. Sjöström et al. [29] also described higher values of benzopyrene isomers on the skin of the wrists than on the neck of exposed fire starters (0.35 versus 0.08 ng/cm^2^), smoke divers (0.18 versus 0.05 ng/cm^2^), and team leaders (0.09 versus 0.05 ng/cm^2^). Moreover, Fernando et al. [45] reported dermal concentrations of benzopyrene isomers (0.64 ng/cm^2^), with increased levels on the skin of the forehead (0.20 ng/cm^2^); similar values (0.11 ng/cm^2^) were found on the remaining skin regions (neck, wrist, back, and fingers).

Other possible/probable carcinogenic PAHs were found on the skin of the firefighters after fire combat. Naphthalene was determined in two studies, at levels varying between 1.11 ng/cm^2^ and 25.6 ng/cm^2^ on the neck skin of subjects participating in training exercises [43,45]. Levels of benz(a)anthracene were reported in four studies, with values ranging from 0.04 ng/cm^2^, on the collarbone skin of team leaders (firefighters controlling the fire from outside the burn house; Sjöström et al. [29]), to 33.69 ng/cm^2^, on the neck skin of subjects attending a wood pallet fire [43]. Moreover, Andersen et al. [43] highlighted that benz(a)anthracene was the predominant PAH with carcinogenic properties on the skin of firefighters. In the study performed by Banks et al. [42], the skin values of benz(a)anthracene and chrysene are reported collectively: 0.038 ng/cm^2^ (at the wrist) and 0.050 ng/cm^2^ (at the neck) after a diesel pan fire, and 0.25 ng/cm^2^ (at the neck) and 0.51 ng/cm^2^ (at the wrist) after a particleboard fire. So far, only four studies have described the levels of chrysene on the skin of exposed firefighters, with concentrations ranging between 0.04 ng/cm^2^ on the collarbone skin of team leaders [29] and 3.06 ng/cm^2^ on the neck skin of subjects participating in a training fire [43]. Concentrations of benzofluoranthene isomers, benzo(b)fluoranthene, benzo(j)fluoranthene, and benzo(k)fluoranthene have been reported on the skin of firefighting forces [29,42,45]. The highest levels of benzo(b)fluoranthene was found on the skin of fire starters’ wrists (total of 0.41 ng/cm^2^ [29]), while 0.03 ng/cm^2^ of benzo(j)fluoranthene was the lowest value determined on the skin of the neck, forehead, back, and fingers of firefighters participating in a training exercise with untreated wood as fuel [45]. Recently, Banks et al. [42] also included the analysis of benzo(b)fluoranthene and benzo(k)fluoranthene and observed predominantly increased skin concentrations after particleboard fires (0.33 ng/cm^2^ for the neck and 1.00 ng/cm^2^ for the wrists) than after a diesel-based fire (0.47 ng/cm^2^ on the neck and 0.069 ng/cm^2^ on the wrists). Baxter et al. [34] also highlighted the predominance of benzofluoranthene isomers on the skin of the face and neck of North American firefighters after five fire events. Dermal concentrations of indeno(1,2,3-c,d)pyrene varied between 0.04 ng/cm^2^, on the skin of the collarbones of firefighters working at the fire front [29], to 5.15 ng/cm^2^, on the neck of the subjects involved in a fire with wood pallets, mattresses, and electrical cords [43]. Regarding dibenz(a,h)anthracene, only two studies were able to quantify the levels of this compound on the skin of the firefighters. Banks and coworkers [42] reported values of 0.14 ng/cm^2^ of dibenz(a,h)anthracene on the wrists of firefighters who fought a particleboard fire; this compound was not detected on the neck of those subjects. Sjöström et al. [29] reported lower levels of this PAH (0.06 ng/cm^2^) on the neck of fire starters after participation in a training exercise. The available literature demonstrates the contamination of firefighters’ skin with PAHs released during firefighting activities regardless of the use of PPE. Most of the studies reported the use of PPE during fire combat; however, it is known that firefighters tend to partially remove their protective gear when the fire is under control, including during the overhaul phase, and/or when a reduced risk of exposure to fire emissions is predicted. Recent studies suggest the use of particulate-blocking hoods as a more effective piece of firefighter PPE to reduce the exposure on the neck and face of the subjects [47,48]; however, more studies are warranted. Dermal exposure to fire emissions may also occur due to the limitations of PPE design and the equipment’s fit on the body of the firefighters [26]. Additionally, post-fire cleaning measures as well as regular PPE maintenance procedures vary widely, which will also contribute to the contamination of firefighters’ skin [49,50,51].

## 4. Dermal In Vitro/In Vivo Studies

Once on the human skin, PAHs can be transdermally assimilated through passive diffusion and/or absorption and be metabolized by cytochrome P450 enzymes (CYP450). Different in vitro/in vivo studies performed with animal and/or human skin models have characterized the PAHs’ permeation levels, transdermal flux rates, dermal absorption and bioavailability, and association with toxicological and carcinogenic risks.

### 4.1. Dermal Absorption

The dermal absorption of PAHs is dependent on their transdermal diffusion and/or absorption rates, followed by metabolization via CYP450 monooxygenase in epidermic cells and their conversion into more hydrosoluble compounds [52]. Nevertheless, the diffusion through the nonviable stratum corneum, the outer skin layer, is the rate-limiting step in the dermal absorption process. Once absorbed, PAHs and/or their metabolites can reach the blood circulation in the dermal layer and be distributed through other tissues and organs of the human body [52]. The rate and extent of dermal absorption are important parameters for the determination of the total internal dose of PAHs dermally absorbed [53].

Table 1 summarizes the available information related to the dermal absorption of PAHs. Some studies were conducted in animal models (pig [13], guinea pig [54,55], rat [54], and monkey skin [56]), while others were performed in human skin models [52,53,56,57]. A model based on synthetic human skin, with sweat and sebum, was described by Luo et al. [14].

Roy and coworkers used structure activity relationship models based on the partition coefficient and the percentage of the applied dose dermally absorbed to estimate the dermal absorption of the different PAHs [58,59]. The results obtained by the authors suggest that low-molecular-weight PAHs presented increased dermal absorption rates (37 ng/cm^2^/h for fluorene, 24 ng/cm^2^/h for naphthalene, 20 ng/cm^2^/h for phenanthrene, and 11 ng/cm^2^/h for acenaphthene) compared to the values reported for high-molecular-weight compounds (1.1 ng/cm^2^/h for pyrene, 0.23 ng/cm^2^/h for benz(a)anthracene, 0.016 ng/cm^2^/h for benzo(a)pyrene, and 0.0013 ng/cm^2^/h for indeno(1,2,3-cd)pyrene) (Table 1). These findings are in line with the results reported by other authors who also observed that low-molecular-weight PAHs are more easily absorbed by the skin and have increased dermal permeabilities than heavier compounds [13,14,57]. Moody et al. [57] reported that naphthalene, phenanthrene, fluoranthene, pyrene, benz(a)anthracene, chrysene, and benzo(k)fluoranthene presented dermal absorption rates predominantly above 80% in synthetic human skin and human breast skin (Table 1). For other PAHs, lower total dermal absorption rates were described (e.g., benzo(a)pyrene, benzo(b)fluoranthene, dibenzo(a,h)anthracene, benzo(g,h,i)perylene, and indeno(1,2,3-c,d)pyrene) (Table 1). Regarding studies performed in animals models, Alalaiwe et al. [13] and Sartorelli et al. [56] reported similar values for dermal naphthalene permeability rates in pig and monkey skin (≈5–6 × 10^−3^ cm/h), respectively. Similar permeability rates were also described for acenaphthene and fluorene (6.33 × 10^−3^ cm/h and 6.26 × 10^−3^ cm/h, respectively) (Table 1).

The dermal absorption of PAHs is affected by the physicochemical and structural properties of compounds. Sartorelli et al. [56] highlighted the importance of considering the octanol/water partition coefficient (K_OW_) in skin models to better predict the permeability constant of each compound. These findings are supported by the results described by other authors [13]. PAHs with high molecular weights (>200 g/mol) and log K_OW_ presented lower dermal absorptions and skin permeability flux [13,14,53,56,57]. Ng et al. [55] concluded that the dermal absorption of phenanthrene is predominantly controlled by passive diffusion rather than by metabolism. In addition, some other factors need to be taken into consideration during dermal absorption studies, including the site of contamination application, the selected animal species, the temperature employed during the assay, the levels of skin hydration, and the solubility of the analyte [55]. A radioactive species of benzo(a)pyrene was used to measure the recovery of this carcinogenic PAH after 48 h of exposure and the rate of skin permeation in different animal tissues and human skin [54]. It was found that rat and guinea pig skin presented increased dermal permeation rates (maximum values of 0.38 and 0.42 µg/cm^2^/h, respectively) compared to the abdomen skin of 32- and 50-year-old individuals (0.02 and 0.01 µg/cm^2^/h, respectively) (Table 1).

Concerning studies evaluating the dermal absorption of PAHs over time, Luo et al. [14] reported that the permeability of four PAHs (naphthalene, phenanthrene, pyrene, and benzo(a)pyrene) on human synthetic skin reached an equilibrium state after 3 to 6 h post-exposure to low (5 µg/L) and high (10 µg/L) concentration values; a decrease on the permeability rates was observed up to 9 h post-exposure. The nature and duration of exposure are important parameters that influence the dermal absorption of PAHs. Higher exposure levels can contribute to the enhanced deposition of PAHs on the skin, which may result in increased dermal absorption fluxes. However, when the skin saturation level is reached, lower penetration rates can be observed [52]. Bourgart et al. [52] found that the fraction of total unmetabolized benzo(a)pyrene globally decreased with the exposure time, regardless of the dose applied. The authors also determined the levels of three benzo(a)pyrene metabolites, benzo(a)pyrene-tetrol, benzo(a)pyrene-7,8-diol, and 3-hydroxybenzo(a)pyrene, in ex vivo human breast skin, as well as in the culture medium used (DMEM/F12, a common culture medium for the growth of mammalian cultures). The concentrations of all metabolites increased over time, being only detectable 24 h post-exposure, which was related with the steps involved in the metabolic pathway [52]. Moreover, the percentage of unmetabolized benzo(a)pyrene remaining on the skin surface after application was reduced with increasing exposure time.

Regarding the selection of the model (animal versus human skin), other factors should be taken into consideration. Due to the restrictions of animal use, ex vivo human skin models are preferable and have demonstrated a satisfactory correlation between in vivo and in vitro studies. Ex vivo samples from freshly excised human skin serves as the best representative model to reproduce realistic cutaneous exposure in humans, since it eliminates interspecies extrapolation and overestimates the dermal penetration often observed with animal models [52]. Reduced costs as well as faster results are other positive aspects [55]. However, the limited access to in vivo/ex vivo human skin has motivated the choice of animal models (e.g., mice, rat, or guinea pigs). Moreover, studies using human skin tissues have some limitations that should be considered during assay planification. The applied dose has a great influence on the results, which could affect the use of in vitro data on in vivo models for human risk assessment. The inter-individual variability of different skin donors (e.g., ethnicity, gender, genotype, general health, local blood flow, and formation and duration of skin depot) will also impact the results. In a realistic scenario, the dermal exposure comprises skin contact with complex mixtures containing several PAHs, among other pollutants. Therefore, both human and animal in vitro/in vivo studies involving a limited number of pre-selected PAHs could bias the effective dermal absorption rates.

### 4.2. Dermal Bioaccessibility and Bioavailability

The dermal bioaccessible fraction of PAHs represents the amount of topical PAHs that are bioavailable to be assimilated by the skin and to be subjected to cutaneous metabolic processes, while dermal bioavailability refers to the fraction of bioaccessible PAHs that can reach systemic circulation and the specific tissues and organs of the human body and exert its biological effects. Unmetabolized PAHs are poorly bioavailable after dermal exposure, which is related to enhanced first-pass metabolism, conditioning the carcinogenicity of unmetabolized PAHs to the local contamination on the skin. PAH metabolites easily cross the human skin, since they are more polar and have a superior hydro-solubility than the respective unmetabolized compound [52].

So far, few studies have explored PAHs’ dermal bioavailability through in vitro/in vivo assays performed with animal models and human skin tissues. Firstly, Kadry et al. [60] resorted to radioactive species to compare the oral and dermal bioavailability of phenanthrene, and reported slower dermal absorption (8.6 h versus 1.0 h half-life time, respectively) and faster elimination (16.1 h versus 28.0 half-life time) than gastrointestinal tract in rats. These findings are in line with previous studies [61,62] reporting the elimination of PAHs after only 8 h of exposure in rats [60]. After 96 h of post-dermal exposure to radioactive phenanthrene, the skin application site presented the highest levels of radioactivity (0.310–0.840% initial dose/g tissue), followed by the ileum (0.021–0.046% initial dose/g tissue) and duodenum (0.017–0.021% initial dose/g tissue); radioactivity was also found in untreated skin (0.007–0.023% initial dose/g tissue) [60]. Kadry and coworkers [60] explained the distribution of radioactivity to the intestinal tissues as being due to the biliary excretion of phenanthrene metabolites, which are later eliminated through the urine as phenanthrenequinone, 9,10-phenanthrenedihydrodiol, phenanthrol, and phenanthrene. The authors were able to demonstrate that, after dermal exposure, phenanthrene become significantly available to skin tissues.

Some authors explored the dermal bioavailability of PAHs due to the exposure to contaminated soils [63]. Different skin absorption experiments were performed with human skin exposed to soils contaminated with PAHs during controlled periods, with the receptor fluids collected at different post-dose moments. The in vitro dermal absorption revealed a slow release/diffusion of benzo(a)pyrene (50–300 pg/cm^2^/h) and reached a steady-state flux within 48–98 h after dermal contact [63]. Roy et al. [58] reported a close range, but slightly higher benzo(a)pyrene dermal flux rates (0.0064 to 750 ng/cm^2^/h) also reached a stationary state of diffusion 48 h post-exposure. The available evidence suggests that absorbed dermal rates only represent a small fraction of the total dose applied (maximum reported value of 12%), thus validating a stationary permeability state with the absorption of a constant concentration over time [63].

The dermal absorption fraction can be used to express the dermal bioavailability. Recently, Forsberg et al. [64] calculated the dermal absorption fraction for six PAHs in the skin of female mice 24 h post-exposure to soils contaminated with PAHs and reported mean values of 0.85–3.6% for benzo(b)fluoranthene, 0.52–1.4% for benz(a)anthracene, 0.58–1.3% for benzo(a)pyrene, non-detected to 0.46% for chrysene, and non-detected to 0.42% for benzo(k)fluoranthene; dibenz(a,h)anthracene was not detected. Some authors highlighted that human skin is less permeable to chemical substances, including PAHs, than the skin of primates [64,65]. Additional research is needed to characterize the dermal bioavailability of PAHs in human skin tissues.

### 4.3. Toxicological and Carcinogenic Dermal Risks

Most PAHs require bioactivation through a complex enzymatic metabolism to be biotransformed into reactive intermediates that might cause mutagenicity, teratogenicity, and carcinogenicity in targeted biological molecules (e.g., DNA and proteins). PAHs can activate AhR and promote the gene expression of metabolizing enzymes such as CYP450 (e.g., CYP1A1, CYP1A2, and CYP1B1) [23]. These enzymes catalyze the oxidation of pro-carcinogens to carcinogenic reactive intermediates, and it is believed that this is the first step in the metabolism of PAHs that will promote the activation of the carcinogenicity process. The predominant mechanism involved in the activation of PAHs carcinogenesis results from mutational miscoding caused by the enzymatic generation of sterically hindered bay- or fjord-region diol epoxides by epoxide hydrolase that will generate DNA adducts [66,67]. For benzo(a)pyrene, a potent skin carcinogen in different animal species, the ultimate carcinogen is the bay-region diol epoxide benzo(a)pyrene-7,8-dio-9,10-epoxide [66]. Other relevant mechanisms in the activation of carcinogenicity are the formation of radical cations (e.g., by electron oxidation via peroxidases) and/or the generation of *o*-quinones via dihydrodiol dehydrogenases [66,68,69]. The metabolism of PAHs causes the activation of AhR and is directly associated with alterations in cell signaling and proliferation, which will promote oxidative stress and thus contribute to the cells’ toxicity and carcinogenicity [66,70]. Dermal exposure to PAHs contributes to local inflammation, atopic dermatitis, skin aging, and skin diseases, including cancer [8,9,10].

Studies assessing the toxicological and carcinogenic risks of PAHs on skin tissues have been slowly emerging in the literature (Table 2). Sivak et al. [71] evaluated the incidence of sores and scabs after the skin of mice had been exposed to different combinations of condensed roofing asphalt fumes, including three fractions containing known levels of benzo(a)pyrene (0.01%, 0.001%, and 0.0001%). The bioassay consisted of the dermal administration of the tested materials twice a week, for 104 weeks, on 40 groups of 30 mice. The strongest response was found after the treatment with the highest concentration of benzo(a)pyrene (0.01%), which affected the sensitivity to assess the cocarcinogenic activity due to a saturation effect. Overall, a significant number of tested individuals died (all for the highest level of benzo(a)pyrene contamination) and the presence of tumors and carcinomas was found in several animals (Table 2). When the treatment was performed with the lowest concentration of benzo(a)pyrene (0.0001%), the presence of tumors or carcinomas was not observed, although 50% of individuals also died [71]. Hall et al. [72] studied the stereoselectivity mechanism of benzo(a)pyrene in human skin obtained from reduction mammoplasty, mastectomy, or amputation through the determination of dihydrodiols and tetrols, including the enantiomeric composition of the four benzo(a)pyrene tetrols, since the stereoselectivity of benzo(a)pyrene may be an important factor for carcinogenicity. Different intermediates were found on skin extracts, with benzo(a)pyrene-(7,8)-dihydrodiol being the predominant compound (1.71–18.27 pmol/cm^2^ skin), mostly in the form of enantiomer (-)-7R,8R (Table 2).

These findings confirm the metabolic activation of benzo(a)pyrene [72]. The concentrations of benzo(a)pyrene tetrols ranged between 2.1 and 10,230 pmol/cm^2^ skin (Table 2).

Hall and coworkers [60] observed variations among individual mice in the stereoselective metabolism of benzo(a)pyrene, which will have implications for the individual’s susceptibility to induced skin carcinogenesis due to the dermal contact with PAHs. More recently, Bourgart et al. [52] studied the metabolism of benzo(a)pyrene when higher, but realistic, doses (up to 22.11 nmol/cm^2^) of this carcinogenic PAH were applied to an ex vivo skin model prepared with freshly excised human breast skin. After 8 h post-exposure, benzo(a)pyrene vastly penetrated in the skin, with 3-hydroxybenzo(a)pyrene and 7,8,9,10-tetrahydroxy-7,8,9,10-tetrahydrobenzo(a)pyrene being the predominant metabolites found in the culture medium. The dermal absorption and metabolization of benzo(a)pyrene continued to occur over 48 h after topical application (Table 1). The dermal bioavailability of benzo(a)pyrene was limited, and the accumulation of unmetabolized benzo(a)pyrene in and on the skin as the applied dose increased during the biological assay; less than 3% of the applied dose remained in the culture medium (Table 1). Bourgart and coworkers [52] found that the benzo(a)pyrene tetrol metabolic pathway was rapidly saturated as the applied dose increased, probably due to the saturation of epoxide hydrolase [52]. The authors highlighted that benzo(a)pyrene carcinogenicity is located on the contaminated skin tissue, since unmetabolized benzo(a)pyrene is poorly bioavailable after dermal exposure due to an enhanced first-pass metabolism mediated by CYP450 and epoxidehydrolase. Alalaiwe et al. [13] explored the absorption of six PAHs (naphthalene, fluoranthene, pyrene, chrysene, benz(a)anthracene, and benzo(a)pyrene) through the dermal route and estimated their effect on the cutaneous inflammation of pig dorsal skin. The dermal deposition of PAHs increased with a superior lipophilicity and molecular size of compounds, while skin absorptions were heavily affected by the structures and physicochemical properties of the PAHs. Compounds with higher dermal absorption rates presented stronger interactions with stratum corneum lipids [13]. After in vitro skin permeation, cultured keratinocytes were used to determine the cytotoxicity and increased levels of cyclo-oxygenase-2, prostaglandin E2, chemokine (C-X-C motif) ligand 1, and interleukin-8 were released by exposed cells [13]. Benzo(a)pyrene, benz(a)anthracene, and chrysene caused the highest expression in the concentrations of cyclo-oxygenase-2 and prostaglandin E2, while naphthalene (2-ring PAH) promoted the lowest expression of these biomarkers (Table 2). Alalaiwe and coworkers [13] were able to demonstrate that particulate matter containing PAHs caused stronger cytotoxicity as well as skin barrier deficiency via the inhibition of the filaggrin and integrin β1 expression (markers for the formation of skin barrier function) than particles containing heavy metals. Therefore, the permeation of PAHs into and across the animal skin was proven. These authors found a strong in vivo/in vitro correlation, with benz(a)anthracene and benzo(a)pyrene having a great potential to disrupt the skin barrier and to cause enhanced inflammation in in vivo models [13].

Potratz et al. [66] studied diverse signaling pathways related to the dermal exposure to chrysene, benzo(a)pyrene, and dibenzo(a,l)pyrene through the determination of several endogenous metabolites. This metabolomic approach was performed on an in vitro assay with human adult wildtype (HaCat WT) and AhR knockdown (HaCaT AHR) keratinocyte cell lines. The authors reported changes in the levels of 24 metabolites (including biogenic amines, acylcarnitines, amino acids, phosphatidylcholines, and sphingomyelines), which represent alterations in the regular metabolic profile of the cell lines, suggesting the influence of PAHs on the energy and lipid metabolism [66]. More specifically, lower concentrations of the amino acid glutamine were found after the cell lines’ exposure to benzo(a)pyrene and dibenzo(a,l)pyrene. However, Potratz et al. [66] highlighted that the depletion of amino acids can result from the glutathione synthesis. PAHs are lipophilic and can incorporate cellular membranes and disturb its biological function. Strong phosphatidylcholine changes were suspected to be associated with perturbations of membrane integrity [66]. Moreover, Potratz et al. [66] reported increased values of some sphingomyelines and associated these findings with the onset of apoptosis in the analyzed cell lines 48 h post-exposure [66]. The enzymes CYP1A1 and CYP1B1 catalyze the oxidation of benzo(a)pyrene and dibenzo(a,l)pyrene, respectively [66,71]. Cell lines exposed to chrysene presented strong cytotoxic effects (with EC50 values of 3.8 and 2.0 µmol/L for HaCaT WT and HaCaT AHR cells, respectively), while the treatment with dibenzo(a,l)pyrene enhanced the cytotoxicity in HaCaT WT cells (EC50 of 0.0035 µmol/L); the exposure to benzo(a)pyrene caused limited cytotoxicity (Table 2). Cell lines exposed to carcinogenic PAHs at levels capable of activating AhR demonstrated a strong capacity to enhance the prototype death receptor CD95, the best-characterized member of the tumor necrosis factor superfamily of receptors [74]. Potratz et al. [66] successfully demonstrated the activation of AhR by PAHs and the consequent induction of CYP1A1 and CYP1B1 at the mRNA and protein levels. Their expression was significantly enhanced in cells treated with the highest levels (3.5 µmol/L) of benzo(a)pyrene and chrysene. Cell lines treated with dibenzo(a,l)pyrene, a potent carcinogen in mice, registered a considerable lack of gene induction; however, the concentrations of the formed metabolites remained virtually unaffected [66]. A possible explanation for these findings relies on the fact that HaCaT AHR might become insensitive and partially/totally lose their capacity to differentiate dibenzo(a,l)pyrene. As CYP1B1 is mainly responsible for the metabolic activation of this PAH, a decrease in the cellular level of this enzyme might explain the reduced response of HaCaT AhR against the potential toxicity of this compound. Moreover, the dibenzo(a,l)pyrene’ AhR-dependent toxicity could be caused by the non-genotoxic AhR signaling alone. Despite modifications to the AhR target genes, CYP1A1 and CYP1B1, Siddens et al. [73] determined the changes in other genes of expression in the skin of mice. After 12 h post-treatment with PAHs, the exposure to benzo(a)pyrene caused the formation of three-fold change levels of total DNA adducts, biomarkers of predicted carcinogenicity, than dibenzo(a,l)pyrene (141 versus 45 adducts/108 nucleotides) (Table 2). The formation of PAH-DNA adducts in the skin is not sufficient to predict the final tumor response. Siddens et al. [73] observed and registered the tumor incidence on a weekly basis with 25-week promotion intervals after mice dermal exposure to benzo(a)pyrene and dibenzo(a,l)pyrene. The authors demonstrated that dermal exposure to benzo(a)pyrene and dibenzo(a,l)pyrene predominantly produced papillomas, followed by squamous cell carcinoma and in situ carcinoma in mice. The authors also highlighted the greater carcinogenicity of dibenzo(a,l)pyrene than the predicted values, based on the available relative potency factors [73].

Recently, Beitel et al. [32] used an in vitro PAH CALUX^®^ bioassay to assess the overall AhR-mediated toxicity of PAHs present on dermal wipe samples and the urinary PAH metabolites collected by North American firefighters before and after participation in a controlled fire. The authors found greater concentrations of AhR-active compounds on the neck and calf of firefighters’ post-fire, which demonstrates the deposition of PAHs and other AhR active compounds on the skin. These findings are aligned with the reported levels of total PAHs on the skin of exposed firefighters (Figure 1). This study successfully proved that the post-fire cleaning of the head and neck area with a baby wipe significantly decreased the majority of the AhR-active compounds on the skin of firefighters, contributing to a reduction in the time of dermal exposure to fire emissions. PAHs are photo-reactive and phototoxic airborne pollutants and, therefore, the simultaneous dermal exposure to sunlight and PAHs might have synergetic effects on the skin. Soeur and coworkers [75] studied the combined influence of dermal daily UV irradiation and exposure to benzo(a)pyrene and indeno(1,2,3-c,d)pyrene on a reconstructed skin model based on human epidermal keratinocytes. The cell viability, clonogenic efficiency, glutathione metabolism, and redox homeostasis were evaluated. The authors observed that low concentrations of PAHs caused high cellular stress, phototoxicity, and impaired keratinocyte clonogenic potential (subtoxic levels), as well as the generation of ROS and decreased intracellular glutathione levels within the cells after several hours post-exposure [75]. The effect caused by benzo(a)pyrene was increased in comparison to the response caused by topical indeno(1,2,3-c,d)pyrene [75]. Soeur et al. [75] highlighted that people experiencing photo-pollution stress (e.g., firefighting forces) may have alterations in their cutaneous homeostasis and promote skin damage and aging. Regarding urine samples, Beitel and coworkers [32] found increased values of AhR-active compounds after 2–4 h of post-firefighting and enhanced potencies (compared to benzo(a)pyrene) were observed for some PAH metabolites: 3-hydroxychrysene (relative potency EC20 and EC50 of 4.30 × 10^−3^ and 4.43 × 10^−3^, respectively), 4-hydroxyphenanthrene (5.78 × 10^−4^ and 5.89 × 10^−4^), 3-hydroxyfluorene (4.44 × 10^−4^ and 5.41 × 10^−4^), 6-hydroxychrysene (3.45 × 10^−4^ and 3.40 × 10^−4^), 2-hydroxynaphthalene (2.99 × 10^−5^ and 6.08 × 10^−5^), and 2-hydroxyphenanthrene (2.69 × 10^−5^ and 5.63 × 10^−5^); 1-hydroxypyrene had a relative potency EC20 of 9.70 × 10^−6^ (Table 2). A correlation was found between the bioassay response and the urinary levels of PAH metabolites; however, the values of increased AhR-active compounds in post-fire urine were probably also due to the presence of other PAH-related compounds [32]. Unsubstituted, nitro, and methylated PAHs, known mutagens and/or carcinogens, also presented AhR-mediated activity, some with expected superior potency than parent PAHs [76]. Therefore, more studies evaluating the potential toxicological and carcinogenic risks caused and/or promoted by dermal exposure to PAHs are needed.

## 5. Conclusions

Information related to firefighters’ dermal exposure to PAHs released during fires remains limited. A lack of uniformity was marked in the sampling and analytical methods used by different authors. However, the available data clearly demonstrate the presence of PAHs included in the United States Environmental Protection Agency’s list of priority pollutants on the skin of exposed firefighters, even when the adequate use of firefighting PPE was reported. The neck, wrists, and face were the primary contaminated body regions, regardless of the use of PPE. Some PAHs were also detected on the calf, scrotum, and back of exposed firefighters. These findings are somewhat expected, since these are the less-protected areas by the PPE, mostly due to the limitations of equipment design and the pre-cleaning procedures.

The available in vitro/in vivo assays demonstrate that PAHs can reach deep skin layers, even at low concentrations, either due to passive diffusion and/or absorption into the epidermis, with further metabolization and systemic distribution to other tissues and organs of the human body. PAHs with low molecular weight present increased dermal absorption rates than high-molecular-weight compounds, being more easily absorbed by skin tissues and, thus, presenting increased topical permeabilities. There are several factors that affect the dermal absorption of PAHs, e.g., the physicochemical and structural properties of compounds, nature and duration of exposure, and factors related to the skin cell lines used in the assay (hydration, animal species, etc.). However, PAHs’ dermal absorption and bioavailability rates remain poorly characterized, mainly in human skin tissues. Evidence highlights the potential of dermal exposure to PAHs to promote cytotoxicity, oxidative stress, topical inflammation, and to activate the carcinogenic process through the activation of AhR and the consequent enzymatic generation of reactive intermediates, that will generate protein and/or DNA adducts. The generated reactive intermediates will be transformed via CYP450 enzymes to expedite their excretion from the organism as PAH metabolites in biological fluids (e.g., urine, bile, feces, and milk) [2]. Firefighters’ regular dermal exposure to PAHs promotes the development and/or aggravation of skin disorders, with limited evidence suggesting increased values of AhR-mediated toxicity and urinary PAH metabolites after participation in fires. Future research needs to continue characterizing the dermal absorption and bioavailability of PAHs and the associated toxicological and carcinogenic effects. The use of bioavailable concentrations, rather than dermal exposure levels, will allow a more realistic health risk assessment of human exposure to PAHs. The contribution of dermal exposure to the total internal dose of PAH remains unclear. Therefore, more studies are needed to explore the relation between the topical values of PAHs, their bioavailability, and the levels of their metabolites in biological fluids. The missing data will be determinant to define maximum levels for dermal exposure to PAHs, which is of extreme importance in heavily occupationally exposed groups such as firefighting forces. It will also allow the evaluation of the preventive measures, e.g., the decontamination of skin and PPE procedures already implemented by some fire brigades, to minimize firefighters’ occupational exposure through dermal contact.

## Figures and Tables

**Table 1:** Data collected from in vitro/in vivo studies assessing dermal absorption of PAHs.

Model (Dosing Vehicle)	PAHs	Units	Results	Main Conclusions	Reference
Hairless guinea pig (acetone)	Phe	Mean ± SEM (%)	(a) Permeation in vitro flow-through cells (balanced salt solution, 6.6 µg/cm^2^) Receptor fluid: (6 h) 2.4 ± 0.5; (12 h) 6.4 ± 1.2; (18 h) 8.3 ± 1.3; (24 h) 8.7 ± 2.0 Dose remaining in skin: 59.7 ± 2.0; Total dose: 68.5 ± 2.0; Skin wash: 10.3 ± 2.0; Recovered dose: 78.8 ± 2.1 (b) Permeation in vitro flow-through cells (balanced salt solution, bovine serum albumin, 6.6 µg/cm^2^) Receptor fluid:(6 h) 38.9 ± 8.1; (12 h) 63.6 ± 8.8; (18 h) 73.4 ± 8.5; (24 h) 78.0 ± 8.6 Dose remaining in skin: 11.8 ± 0.1; Total dose: 89.7 ± 8.7; Skin wash: 3.5 ± 1.2; Recovered dose: 93.3 ± 8.4 (c) Permeation in vitro flow-through cells (water, bovine serum albumin, 15.2 µg/cm^2^) Receptor fluid: (6 h) 20.8 ± 0.5; (12 h) 41.4 ± 0.9; (18 h) 52.1 ± 2.5; (24 h) 57.4 ± 2.7 Dose remaining in skin: 8.8 ± 1.0; Total dose: 66.2 ± 3.3; Skin wash: 2.2 ± 0.2; Recovered dose: 68.4 ± 3.5 (d) Permeation in vitro flow-through cells (balanced salt solution, bovine serum albumin, 15.2 µg/cm^2^) Receptor fluid: (6 h) 33.7 ± 7.3; (12 h) 58.1 ± 7.7; (18 h) 67.2 ± 6.5; (24 h) 71.3 ± 5.7 Dose remaining in skin: 7.8 ± 2.8; Total dose: 79.1 ± 3.6; Skin wash: 1.6 ± 0.4; Recovered dose: 80.7 ± 3.2	In vitro percutaneous absorption of Phe; Penetration through pig skin controlled more by the passive rate of diffusion than by metabolism.	[55]
Rat, guinea pig, and human abdomen skin (acetone)	BaP	Mean ± SD (%)	(a) In vitro recoveries after topical applications with BaP Activity (radioactive soap, water washes, 24 h): (Rat) 5.3 ± 0.32; (Guinea pig) 45.9 ± 3.95; (Human, 32 y) 74.0 ± 6.94; (Human, 50 y) 42.9 ± 5.05 Activity (methanol extracts, 48 h): (Rat) 21.1 ± 5.53; (Guinea pig) 4.9 ± 1.47; (Human, 32 y) 13.5 ± 2.11; (Human, 50 y) 35.2 ± 8.11 Activity (skin digest): (Rat) 22.9 ± 5.24; (Guinea pig) 18.2 ± 4.16; (Human, 32 y) 6.6 ± 2.55; (Human, 50 y) 7.3 ± 0.81 Activity (methanol extract, skin digest): (Rat) 44.0 ± 10.35; (Guinea pig) 23.1 ± 2.76; (Human, 32 y) 20.1 ± 4.62; (Human, 50 y) 42.5 ± 8.71	BaP is well absorbed through animal and human skin; Assessment of total exposure to BaP should consider the dermal route.	[54]
		(µg/cm^2^)	(b) In vitro cumulative absorption of BaP topical application (48 h) (Rat) 5.6 ± 0.10; (Guinea pig) 3.7 ± 0.18; (Human, 32 y) 0.3 ± 0.07; (Human, 50 y) 0.1 ± 0.05		
		(µg/cm^2^/h)	(c) In vitro maximum rate of skin permeation (Rat) 0.38 ± 0.028; (Guinea pig) 0.42 ± 0.031; (Human, 32 y) 0.02 ± 0.009; (Human, 50 y) 0.01 ± 0.003		
		(%)	(d) In vivo total (urinary, fecal, and tissue) dermal absorption of BaP (Rat) 69.4 ± 7.59; (Guinea pig) 67.8 ± 9.33		
Monkey skin (acetone)	8 PAHs	Mean ± SD (cm/h)	Permeability constants Naph: (5.12 ± 2.88) × 10^−3^; Ace: (6.33 ± 4.81) × 10^−3^; Flu: (6.26 ± 4.74) × 10^−3^; Phe: (1.96 ± 1.14) × 10^−3^; Ant: (3.44 ± 3.09) × 10^−3^; Pyr: (1.69 ± 1.36) × 10^−3^; Chry: (0.22 ± 0.14) × 10^−3^; BaA: (0.15 ± 0.08) × 10^−3^	K_OW_ values correlated with the permeability constant (r = 0.90, *p* < 0.001) and the lag time (r = 0.81, *p* < 0.01); A multiple linear regression model between permeability constants, K_OW_, and water solubility was reported (*p* < 0.0001).	[56]
Human back skin (6% aqueous solution of polyoxyethylene 20 oleyl ether)	18 PAHs	Mean ± SD (ng/cm^2^/h)	Directly measured values for flux: Ant 6.5 ± 0.9; Fln/Pyr 1.8 ± 0.3; 3–6 ring PAC: 120 ± 30	High molecular weight compounds presented a reduced dermal penetration flux value.	[53]
		%	Applied dose absorbed: Ant 5.3; Fln/Pyr 3.3; 3–6 ring PAHs: 1.8		
		(ng/cm^2^/h)	Dermal penetration flux values: Naph: 24; Acen: 0.094; Ace: 11; Flu: 37; Phe: 20; Ant: 6.5; Fln: 1.5; Pyr: 1.1; B(a)A: 0.23; Triphenylene: 0.16; Chry: 0.21; B(b)F: 0.035; B(k)F: 0.0044; BeP: 0.062; BaP: 0.016; Ind: 0.0013; DB(a,h)A: 0.0023; B(ghi)P: 0.0075		
Human breast skin (acetone)	11 PAHs	Mean ± SD (%)	In vitro dermal absorption rates in Bronaugh flow-through diffusion cells (24 h skin soap washes) (a) without soil: Phe 88.3 ± 4.83; Fln 82.6 ± 8.09; Pyr 82.5 ± 7.32; BaA 82.3 ± 8.70; Chry 81.5 ± 11.44; B(b)F 77.6 ± 7.49; B(k)F 81.0 ± 9.67; BaP 70.8 ± 5.73; DB(a,h)A 76.7 ± 15.36; B(ghi)P 75.2 ± 9.53; Ind 78.3 ± 7.17 (b) with soil: Phe 60.8 ± 7.75; Fln 49.9 ± 3.70; Pyr 48.7 ± 5.63; BaA 26.4 ± 8.09; Chry 33.5 ± 12.40	High molecular weight compounds presented reduced dermal absorption.	[57]
Synthetic human skin (simulated artificial sweat and sebum mixture)	4 PAHs	Value or Range (µg/L)	Skin absorption rates (up to 9 h post-exposure): Naph 0.22–1.84; Phe 0.24–2.30; Pyr 0.32–0.92; BaP 0.05–0.08 Intracellular levels: Naph 0.26, 0.76; Phe 0.47, 0.92; Pyr 0.45; BaP 0.08, 0.13 Residual levels: Naph 0.61, 1.13; Phe 0.63, 1.45; Pyr 1.13, 1.14; BaP 0.12	Low molecular weight PAHs were more easily absorbed by skin cells than heavier compounds;	[14]
		%	Total dermal penetration: Naph 76.4, 79.9; Phe 72.6, 73.3; Pyr 52.2, 38.7; BaP 8.30, 9.07 Total dermal absorption: Naph 84.6, 87.5; Phe 82.0, 82.5; Pyr 61.2, 43.2; BaP 10.9, 9.87 Loss ratio: Naph 6.19, 1.25; Phe 5.44, 3.00; Pyr 16.2, 46.4; BaP 96.7, 88.9	Dermal permeabilities were increased in 2–3 rings PAHs; Bap metabolism was affected by the levels and duration of exposure and the age of skin.	

SEM—standard error of the mean; SD—standard deviation; K_ow_—coefficient of octanol/water partition; Ace: acenaphthene; Acen: acenaphthylene; Ant: anthracene; BaA: benz(a)anthracene; BaP: benzo(a)pyrene; B(b)F: benzo(b)fluoranthene; BeP: benzo(e)pyrene; B(g,h,i)P: benzo(g,h,i)perylene; B(k)F: benzo(k)fluoranthene; Chry: chrysene; DB(a,h)A: dibenz(a,h)anthracene; Flu: fluorene; Fln: fluoranthene; Ind: indeno(1,2,3-cd)pyrene; Naph: naphthalene; Phe: phenanthrene; Pyr: pyrene.

**Table 2:** Data collected from in vitro/in vivo studies assessing dermal toxicological and carcinogenic risks of PAHs.

Model (Dosing Vehicle)	PAHs	Units	Results	Main Conclusions	Reference
Human breast skin (acetone and tetrahydrofuran)	BaP dihydrodiols and tetrols	Range (pmol/cm^2^)	Concentrations extracted from skin BaP(7,8)-dihydrodiol: 1.71–18.27; BaP(4,5)-dihydrodiol: 0.24–10.43; BaP(9,10)-dihydrodiol: 1.03–12.65; BaP(7,9,10/8) tetrol: 4.6–10230; BaP(7,9/8,10) tetrol: 16.5–2017.7; BaP(7/8,9,10) tetrol: 2.1–630.2; BaP(7,10/8,9) tetrol: 29–1015.8	Observed interindividual variations in the stereoselective metabolism of BaP, which will conditionate the individual susceptibility to PAH-induced skin carcinogenesis.	[72]
Male C3H/HeJ mice (acetone and cyclohexane)	BaP	50th survival (days) Number of death individuals (%) Counts (dimensionless)	Treatment with 0.01% BaP 50th survival: 464; Number of death individuals: 100; Total papilloma/group: 1; Total carcinoma/group: 28; Number of tumors: 27 Treatment with 0.001% BaP: 50th survival: 732; Number of death individuals: 50; Total papilloma/group: 2; Total carcinoma/group: 3; Number of tumors: 5 Treatment with 0.0001% BaP 50th survival: 727; Number of death individuals: 50; Total papilloma/group: 0; Total carcinoma/group: 0; Number of tumors: 0	Groups treated with BaP at 0.01% had such a strong response due to the BaP alone that the sensitivity for assessing cocarcinogenic activity was limited.	[71]
Female FVB/N inbred mice (toluene and 5% DMSO)	BaP, DB(a,l)P	Mean ± SD (adducts/10^8^ nucleotides)	Total DNA adducts BaP: 141 ± 37 DB(a,l)P: 45 ± 13	Exposure produced primarily papillomas followed by squamous cell carcinoma and carcinoma in situ; BaP caused over three times the level of total DNA adducts; DB(a,l)P carcinogenicity was much higher than predicted.	[73]
Human cells—HaCaT cells (0.1% DMSO)	BaP, DB(a,l)P, Chry	EC50 (µmol/L)	Chry EC 50: 3.8; 2.0 DB(a,l)P EC 50: 0.035	Chry caused strong cytotoxic effects in cell lines; BaP and DB(a,l)P up-regulated the levels of metabolites; CYP1A1 and CYP1B1 expression was significantly increased in some cell lines treated with BaP and Chry and the metabolites formed contributed to the observed metabolomic alterations.	[66]
Pig skin (30% propylene glycol/phosphate buffer, pH 7.4)	Naph, Fln, Pyr, Chry, BaA, BaP	(dimensionless)	Toxicological index of PAHs Ciclo-oxigenase-2 Naph: 0.01; Fln: 0.09; Pyr: 0.05; Chry: 0.60; BaA: 0.91; BaP: 0.97 Prostaglandin E2 Naph: 0.07; Fln: 0.34; Pyr: 0.15; Chry: 2.21; BaA: 5.61; BaP: 4.43 Chemokine (C-X-C motif) ligand Naph: 0.34; Fln: 0.81; Pyr: 0.31; Chry: 1.83; BaA: 4.00; BaP: 4.66 Interleukin-8 Naph: 0.08; Fln: 0.19; Pyr: 0.07; Chry: 0.47; BaA: 1.08; BaP: 1.84	BaA and BaP were the compounds revealing the great skin inflammation and barrier function damage.	[13]
CALUX Bioassay (0.8% DMSO)	PAH metabolites	EC20, EC50 (mol/L) REP EC20, EC50 (dimensionless)	Concentrations measured on the assay 1-Hydroxynaphthalene: EC20, EC50 > 2.40 × 10^−4^ 2-Hydroxynaphthalene: EC20: 3.54 × 10^−5^; EC50: 7.50 × 10^−5^ REP EC20: 2.99 × 10^−5^; REP EC50: 6.08 × 10^−5^ 1-Hydroxyphenanthrene: EC20, EC50 > 1.00 × 10^−5^ 2-Hydroxyphenanthrene: EC20: 3.93 × 10^−5^; EC50: 8.11 × 10^−5^ REP EC20: 2.69 × 10^−5^; REP EC50: 5.63 × 10^−5^ 3-Hydroxyphenanthrene: EC20, EC50 > 1.00 × 10^−5^ 4-Hydroxyphenanthrene: EC20: 1.83 × 10^−6^; EC50: 7.75 × 10^−6^ REP EC20: 5.78 × 10^−4^; REP EC50: 5.89 × 10^−4^ 9-Hydroxyphenanthrene: EC20, EC50 > 1.00 × 10^−5^ 2-Hydroxyfluorene: EC20, EC50 > 8.00 × 10^−4^ 3-Hydroxyfluorene: EC20: 2.38 × 10^−6^; EC50: 8.43 × 10^−6^ REP EC20: 4.44 × 10^−4^; REP EC50: 5.41 × 10^−4^ 4-Hydroxyfluorene: EC20, EC50 > 3.00 × 10^−5^ 1-Hydroxypyrene: EC20: 1.09 × 10^−4^; EC50 > 1.00 × 10^−4^ REP EC20: 9.70 × 10^−6^ 3-Hydroxychrysene: EC20: 2.46 × 10^−7^; EC50: 1.03 × 10^−6^ REP EC20: 4.30 × 10^−3^; REP EC50: 4.43 × 10^−3^ 6-Hydroxychrysene: EC20: 3.06 × 10^−6^; EC50: 1.34 × 10^−5^ REP EC20: 3.45 × 10^−4^; REP EC50: 3.40 × 10^−4^ 3-Hydroxybenzo(a)pyrene: EC20, EC50 > 8.00 × 10^−6^; BaP: EC20: 1.06 × 10^−9^; EC50: 4.56 × 10^−9^ REP EC20, REP EC50: 1.00	Increased bioassay response with extracts from post-fire neck and calf wipe samples; Correlation between the bioassay response and urinary levels of PAH metabolites.	[32]

SD—standard deviation. BaA: 1,2-Benz(a)anthracene; BaP: benzo(a)pyrene; Chry: chrysene; DB(a,l)P: dibenzo(a,l)pyrene; Fln: fluoranthene; Ind: indeno(1,2,3-cd)pyrene; Naph: naphthalene, Pyr: pyrene; REP: relative potencies in relation to BaP; DMSO: dimethyl sulfoxide.

## Data Availability

Not applicable.
